# Effectiveness of self-guided virtual reality exposure therapy for social anxiety disorder: a systematic review and meta-analysis protocol

**DOI:** 10.3389/fpubh.2025.1735471

**Published:** 2026-01-12

**Authors:** Yiyuan Li, Jifeng Shen, Guanghui Huang

**Affiliations:** 1 Faculty of Humanities and Arts, Macau University of Science and Technology, Taipa, Macao SAR, China; 2 Macau University of Science and Technology innovation Technology Research Institue, Zhuhai, Guangdong, China

**Keywords:** cognitive behavioural therapy, meta-analysis, self-guided, social anxiety disorder, virtual reality exposure therapy

## Abstract

**Background:**

Social Anxiety Disorder (SAD) is a prevalent anxiety disorder within contemporary mental health practice, attracting increasing attention. Self-guided virtual reality exposure therapy (SGVRET) has been demonstrated to be an effective intervention for SAD in multiple studies. Although research suggests SGVRET may alleviate SAD symptoms, systematic reviews examining its efficacy in treating SAD remain lacking.

**Method:**

This protocol outlines a systematic review designed to assess the efficacy of SGVRET as a intervention for SAD. The study adheres to the Preferred Reporting Items for Systematic Reviews and Meta-Analyses (PRISMA) guidelines. Included studies must be randomised controlled trials employing SGVRET as the intervention. A comprehensive search will be conducted across databases including PubMed, Embase, the Cochrane Library, Web of Science, and Scopus, covering studies from the inception of each database up to September 2025. Data synthesis will be performed via meta-analysis or narrative synthesis based on study homogeneity. Furthermore, Stata version 18.0 software will be employed for meta-analysis.

**Conclusion:**

This review will systematically evaluate the efficacy of SGVRET in social anxiety disorder, providing an evidence base for its standardised application and assessment among individuals with SAD. Findings from relevant studies will also offer actionable guidance for the design, implementation, and optimisation of digital interventions targeting those with social anxiety.

**Systematic review registration:**

https://www.crd.york.ac.uk/PROSPERO/view/CRD420251151820, identifier CRD420251151820.

## 1 Introduction

Social Anxiety Disorder (SAD) is a prevalent anxiety disorder within contemporary mental health practice, characterised by an individual’s excessive fear of potential negative evaluation in social situations, leading to avoidance behaviours and significant psychological distress (1, 2). This social impairment not only affects daily functioning but may also lead to long-term social consequences, including difficulties in employment, interpersonal relationships, and comorbid depression (3, 4). According to the diagnostic criteria of the American Psychiatric Association (DSM-5), the core symptoms of SAD include persistent fear of social interactions, avoidance behaviours, and physiological responses such as palpitations, sweating, and facial flushing (5, 6). Globally, the prevalence of SAD exhibits considerable heterogeneity, yet remains at a relatively high level overall (7). According to relevant studies, the global prevalence of SAD is 4.7% among children, 8.3% among adolescents, and 17% among young adults (8). The lifetime prevalence of SAD is 12.1% (9). The symptoms of SAD can severely impact an individual’s quality of daily life, with affected individuals reporting higher rates of emotional distress, substance misuse, alcohol abuse, and even suicide (10, 11). Moreover, individuals with SAD are susceptible to concerns related to stigma, leading to a tendency to conceal their condition and resulting in many being reluctant to seek healthcare. This not only causes significant personal distress for sufferers but also constitutes a substantial public health economic burden (12–14).

Exposure therapy within cognitive behavioural therapy (CBT) is internationally recognised as the first-line psychological intervention for treating SAD (15, 16), operating by promoting fear extinction and cognitive restructuring through systematic exposure to feared situations (17). However, traditional exposure therapy remains challenging to implement in routine practice, as it relies heavily on real-life scenarios, may provoke heightened fear and stigma leading to increased dropout rates (18), and is constrained by limited therapist availability, particularly in resource-deprived settings (19).

Virtual reality exposure therapy (VRET) has emerged as a digital adaptation of exposure-based cognitive behavioural therapy, enabling patients to engage in controlled exposure exercises within secure, computer-generated environments (20, 21). Multiple meta-analyses have confirmed that VRET demonstrates comparable efficacy to traditional face-to-face CBT for the treatment of SAD (22, 23). In addition, VRET offers numerous advantages, including the capacity to meticulously regulate exposure parameters, such as the number, expressions and behaviours of virtual participants. This capability facilitates the emulation of social scenarios that are challenging to replicate in reality (24). Secondly, VRET also provides a standardised therapeutic experience, and demonstrates good safety and acceptability (25). However, most existing VRET protocols continue to rely on continuous therapist guidance and supervision (26), which constrains scalability and limits broader implementation, particularly in the context of increasing demand for remote and self-directed mental health services following the COVID-19 pandemic.

The advent of self-guided virtual reality exposure therapy (SGVRET) offers an innovative solution to the aforementioned challenges. This model enables patients to complete treatment without professional supervision by embedding the therapist’s guidance functions within the software system (27, 28). This self-guided approach has the potential to enhance accessibility, reduce treatment costs, protect patient privacy, and support more personalised intervention delivery. Nevertheless, SGVRET remains at an early stage of development, and further evidence is required to establish its independent efficacy. Notably, while existing systematic reviews have primarily focused on therapist-guided VRET in SAD, systematic reviews specifically examining the effectiveness of SGVRET in SAD remain scarce (29, 30), limiting its broader application and integration into intervention programmes.

Therefore, this systematic review aims to address this research limitation by synthesising existing evidence on the application of SGVRET for SAD. Specifically, the review has three primary objectives: (1) to evaluate the effectiveness of SGVRET in alleviating social anxiety symptoms, (2) to assess the methodological quality and risk of bias of existing SGVRET studies and examine how study design and intervention characteristics may influence reported outcomes, and (3) to identify key challenges, limitations, and future research directions for the development and implementation of SGVRET. By clarifying the current evidence base, this review may help inform the future development of more accessible digital exposure-based interventions for social anxiety disorder.

## 2 Methods and analysis

### 2.1 Research design registration

This systematic review has been designed in accordance with the Preferred Reporting Items for Systematic Reviews and Meta-Analyses Protocol (PRISMA-P) checklist and the Cochrane Handbook for Systematic Reviews (31, 32). We have registered this systematic review in the International Prospective Register of Systematic Reviews (PROSPERO; CRD420251151820) and reported it following the Systematic Review Declaration. As this constitutes a systematic review, ethical approval is not required.

### 2.2 Procedural roadmap

**Figure 1:**
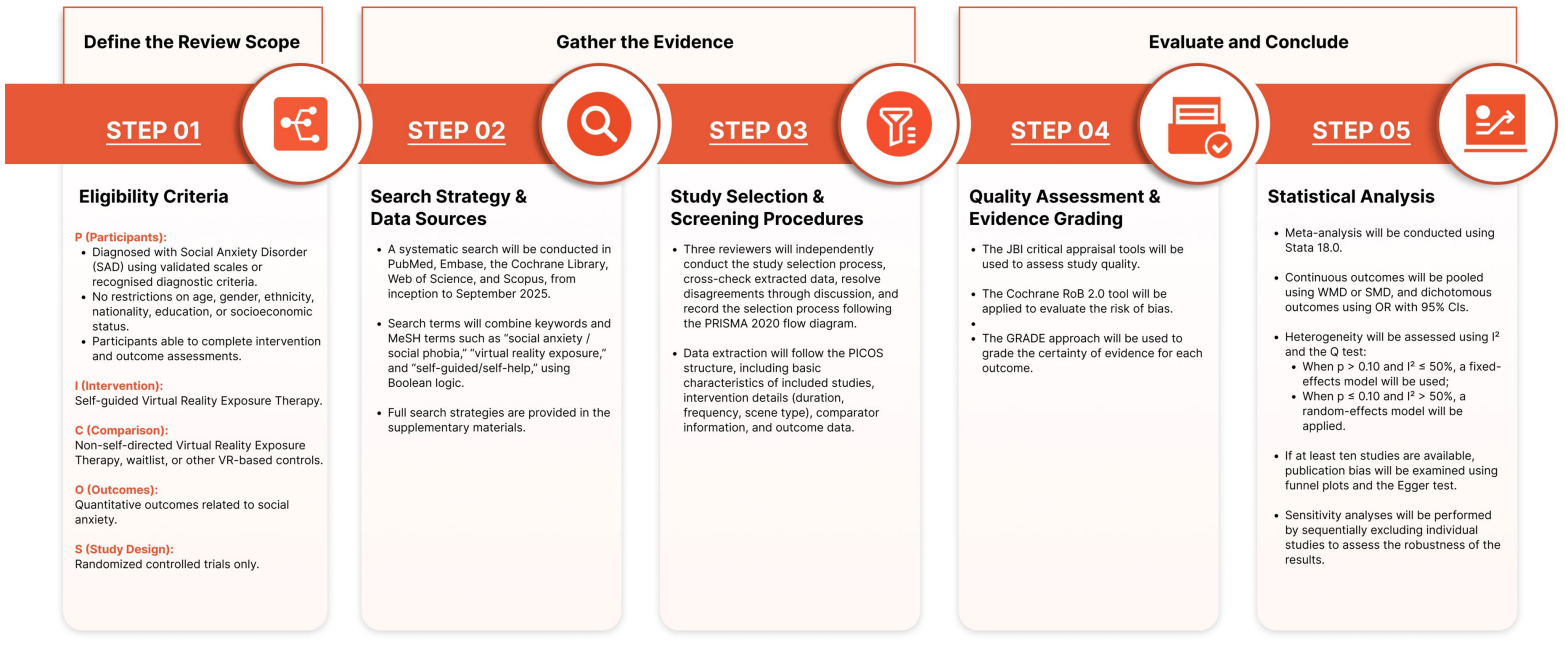
Procedural roadmap.

The procedural framework of this systematic review and meta-analysis will be organised into three sequential phases: Define the Review Scope, Gather the Evidence, and Evaluate and Conclude (Figure 1). The Define the Review Scope phase will correspond to the eligibility criteria and will apply the PICOS framework to delineate the review boundaries, specifying participants diagnosed with social anxiety disorder, self-guided virtual reality exposure therapy as the intervention, non-self-guided VRET or waitlist conditions as comparators, quantitative social anxiety–related outcomes, and randomised controlled trials as the eligible study design. The Gather the Evidence phase will comprise the search strategy and data source identification as well as study selection and screening procedures; systematic literature searches will be conducted across multiple databases, and independent screening and data extraction will be performed by multiple reviewers, with the selection process documented in accordance with PRISMA guidelines and data extraction structured using the PICOS framework. The Evaluate and Conclude phase will encompass quality assessment, evidence grading, and statistical synthesis, in which methodological quality and risk of bias will be assessed using the JBI and Cochrane RoB 2.0 tools, the certainty of evidence will be evaluated using the GRADE approach, and meta-analyses will be conducted to synthesise findings while assessing heterogeneity, publication bias, and the robustness of the results.

### 2.3 Eligibility criteria

The Eligibility criteria for this review adhered to the PICO(S) framework (population, intervention, comparison, outcome, and study design) principles (33), as detailed in Table 1.

**Table 1:** Research screening criteria based on the PICOS format.

PICO(S)	Inclusion	Exclusion
Population (P)	(1) Studies recruiting participants with social anxiety disorder were eligible for inclusion (No restrictions were imposed on age, gender, ethnicity, nationality, educational attainment, or socioeconomic background). (2) Studies were included if participants received a confirmed diagnosis of social anxiety disorder using valid assessment tools, such as the Lieberman Social Anxiety Scale (LSAS), Public Speaking Anxiety Scale (PSAS), Social Interaction Anxiety Scale (SIAS), or other recognised diagnostic criteria. (3)Studies may be included if recruited participants possess the physical capacity to complete interventions and assessments.	Studies that included participants who did not meet diagnostic criteria for social anxiety disorder, those with severe organic pathology or functional impairment, individuals unable to continue observation due to serious physical illness, and subjects with incomplete clinical data.
Intervention (I)	Studies employed self-directed virtual reality exposure therapy as an intervention measure.	Studies not employing self-directed virtual reality exposure therapy as an intervention.
Comparator©	Studies comparing self-directed virtual reality exposure therapy with non-self-directed virtual reality exposure therapy approaches (such as virtual reality exposure therapy or waiting lists).	No exclusion criteria have been applied.
Outcome (O)	Studies containing quantitative outcome data related to social anxiety, such as measurements of anxiety and fear.	Studies lacking quantitative outcome data related to social anxiety, such as qualitative assessment results.
Study Design (S)	Randomised Controlled Trials (RCTs)	Non-randomised controlled trials (such as cohort studies, case–control studies, case reports or review articles)
Other limits	Research studies published in peer-reviewed journals with publication dates ranging from the inception of each database up to September 2025.	Research published outside the stipulated timeframe or literature falling within the category of “grey literature” (such as non-peer-reviewed materials, unpublished manuscripts, or conference abstracts).

#### 2.3.1 Population

This systematic review will include studies that meet the following inclusion and exclusion criteria:

(1) Studies recruiting participants with social anxiety disorder were eligible for inclusion (No restrictions were imposed on age, gender, ethnicity, nationality, educational attainment, or socioeconomic background). (2) Studies were included if participants’ social anxiety was assessed using recognised and validated social anxiety instruments, such as the Liebowitz Social Anxiety Scale (LSAS), Public Speaking Anxiety Scale (PSAS), or Social Interaction Anxiety Scale (SIAS). (3) Studies may be included if recruited participants possess the physical capacity to complete interventions and assessments. Furthermore, participants with severe organic pathology, functional impairment, comorbid mental disorders, or serious physical illnesses rendering them unable to continue participation, or those with incomplete clinical data, shall be excluded.

#### 2.3.2 Intervention

This systematic review focuses on SGVRET as the intervention of interest. SGVRET is operationally defined as a non-pharmacological digital intervention grounded in CBT and exposure principles, in which exposure exercises are delivered through virtual reality systems without real-time, in-session therapist guidance, and is typically self-administered individually, with eligibility determined by the absence of real-time therapist involvement (34). SGVRET is typically delivered via immersive or semi-immersive virtual reality systems that present automated or pre-programmed exposure scenarios, with exposure progression controlled by the system or the participant. Participant interaction is primarily mediated through automated prompts or predefined task structures. Minimal therapist involvement outside the exposure process (e.g., initial guidance, technical setup, or post-session feedback) is permitted, provided that no real-time therapeutic guidance is delivered during the exposure sessions. Accordingly, this review will include studies in which SGVRET constitutes the primary intervention and exclude studies that do not employ a self-guided virtual reality exposure framework as defined above.

#### 2.3.3 Comparator

This systematic review will include studies comparing SGVRET with non-SGVRET conditions (such as VRET or CBT). Additionally, studies comparing combined interventions (such as SGVRET combined with conventional CBT) with alternative therapies used alone (such as VRET alone) will also be included.

#### 2.3.4 Outcome

This systematic review will include studies reporting the following primary and secondary outcomes. Primary outcome measures primarily concern changes in the severity of social anxiety symptoms before and after the intervention. These encompass measurements using validated continuous self-report scales (e.g., LSAS, Libowitz Social Anxiety Scale; PSAS, Public Speaking Anxiety Scale; BFNES, Brief Fear of Negative Evaluation Scale). Additionally, secondary results will encompass changes from baseline to post-intervention and follow-up time points. These alterations include specific domains of social anxiety, such as alongside measurements from scales including the Hospital Anxiety and Depression Scale (HADS) and Patient Health Questionnaire-9 (PHQ-9).

#### 2.3.5 Study design

This systematic review will only include peer-reviewed randomised controlled trials (RCTs). Non-randomised controlled trials, such as observational studies, cross-sectional studies, case–control studies, and qualitative studies, will be excluded.

### 2.4 Data sources and search strategy

The literature search for this study will be conducted systematically across databases including PubMed, Embase, the Cochrane Library, Web of Science, and Scopus. The search scope will cover each database from its inception to September 2025 to ensure inclusion of the most recent relevant studies. Detailed search strategies are provided in Supplementary material 1. To minimise language bias and enhance the global applicability of the research findings, this study imposes no restrictions on the language of publication. Additionally, literature classified as ‘grey literature’, such as non-peer-reviewed articles, unpublished manuscripts, or conference abstracts, will be excluded from the search scope.

### 2.5 Data screening and extraction

#### 2.5.1 Data management and screening

This study implemented a comprehensive literature screening and evaluation process to ensure research objectivity and accuracy. The process will be conducted by three independent reviewers: YYL, JFS, and GHH. Initially, YYL will be responsible for downloading and conducting an initial screening of the literature, excluding studies irrelevant to the research topic. Subsequently, the preliminarily selected relevant literature will be submitted to JFS for a more detailed eligibility assessment. The literature ultimately included in the analysis will be reviewed by both YYL and JFS. Where the two reviewers disagreed, the third reviewer (GHH) made the final decision. Only literature unanimously approved by all three reviewers will be included in the final analysis.

**Figure 2:**
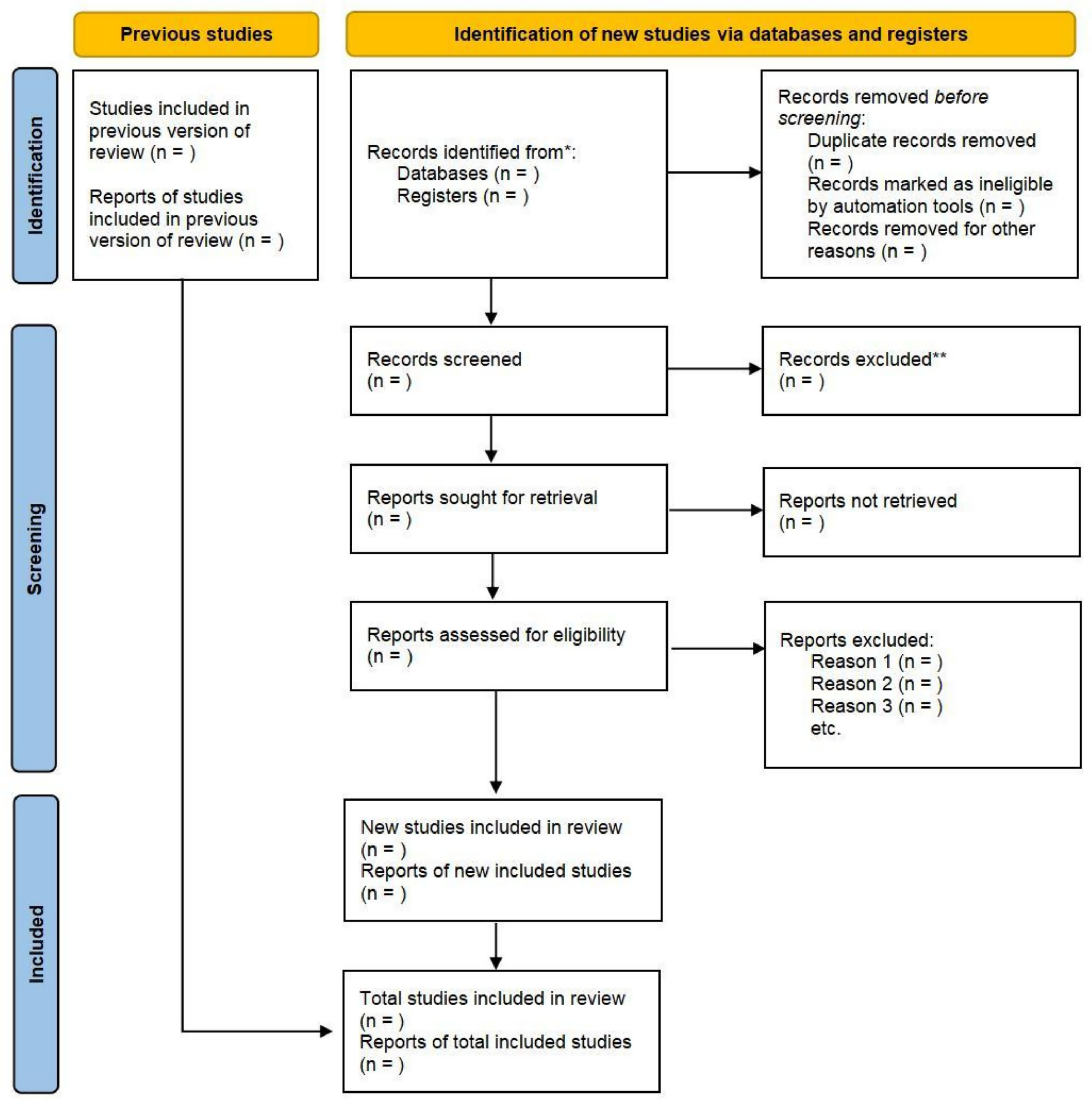
Planned PRISMA 2020 flow diagram for study selection.

This review will assess the full texts of potentially eligible studies to determine the final studies for inclusion. The study screening process will be recorded using a PRISMA 2020 flow diagram. The PRISMA flow diagram is provided in a planned format to illustrate the intended identification, screening, and inclusion process, and study counts will be completed following the literature search and screening (35). The study flowchart is shown in Figure 2.

This figure illustrates the intended study identification, screening, and inclusion process.

Study counts will be populated after completion of the literature search and screening.

#### 2.5.2 Data extraction

Two independent reviewers will use a standardised data extraction form to extract data. A predefined and standardised codebook will be developed to guide the data extraction process, clearly defining each variable and corresponding coding rules to ensure consistency and reproducibility across reviewers (Table 2). During the data extraction process, researchers YYL and JFS will follow the PICO (S) framework, a structured approach for identifying and evaluating the components of clinical evidence in research articles. Specifically, researchers will compile a summary table of study characteristics detailing key information from all included studies, structured as shown in Table 3. This table will encompass the following fundamental elements: (1) Study details (first author, year of publication, country/region of study), (2) Population characteristics (mean age, sample size), (3) Intervention and control (type of intervention, duration of intervention, type of control group), (4) Primary outcomes (measurement tools and primary research findings). In addition to the study characteristics summary, researchers will plan a separate outcome measures summary table to systematically outline all predefined primary and secondary outcomes, their corresponding measurement instruments, and expected assessment time points (Supplementary material 2). When multiple validated social anxiety measures are reported within the same study, selective outcome extraction will be minimised through the application of predefined and consistently applied prioritisation rules. Prioritisation is guided by construct relevance to SGVRET and established psychometric robustness. Specifically, the PSAS will be prioritised in studies primarily targeting public speaking anxiety, given its comprehensive coverage of cognitive, behavioural, and physiological anxiety components and strong validity (36). For overall social anxiety severity, the LSAS will be prioritised due to its direct assessment of fear and avoidance across socially evaluative situations that closely align with exposure-based and virtual reality intervention mechanisms (37). When measures primarily reflect interaction-based or evaluative social anxiety, the Brief Fear of Negative Evaluation Scale (BFNES) or the Social Interaction Anxiety Scale (SIAS) will be selected based on their established reliability and construct validity (38). Where more than one eligible scale is reported, a single outcome measure will be selected according to these predefined criteria to avoid selective outcome extraction bias.

**Table 2:** Predefined and standardised codebook for data extraction.

Data extraction item	Operational definition	Coding rules	Primary source/notes
Intervention duration	Total length of the SGVRET intervention or cumulative exposure	Record total exposure time (minutes) when available; if only weeks/sessions are reported, extract as described; mark as *unclear* if insufficient information	Methods / Intervention
system type	Type of virtual reality technology used	Immersive (HMD-based): head-mounted display; Non−/semi-immersive: desktop-based, screen-based, or video-based systems	Methods (equipment description)
Level of interactivity	Degree of user interaction during exposure	Video-based: passive viewing of pre-recorded scenarios; Interactive: user engagement with tasks, choices, or feedback	Intervention description
Participant characteristics	Baseline characteristics of the study population	Extract baseline symptom severity (scale, mean, SD) and key demographic/clinical features as reported	Baseline / Outcomes
Primary outcome	Main outcome defined by study authors	Prefer the author-defined primary outcome; if not specified, apply a consistent predefined hierarchy	Outcomes
Assessment time point	Timing of outcome assessment	Prioritise the assessment closest to post-treatment; follow-up time points extracted separately	Outcomes

Ambiguous or unreported information will be coded as “unclear” and addressed during analysis.

**Table 3:** Planned structure for the study characteristics summary table.

Author (year)		Country/region	Study design	Sample size (n)	Mean age (years)	Comparator	Outcome	Assessment time points
—	—	—	—	—	—	—	—	—
—	—	—	—	—	—	—	—	—
.	.	.	.	.	.	.	.	.

During data extraction, if outcome data required for synthesis are missing, unclear, or inconsistently reported, the corresponding authors will be contacted to request clarification or additional information.

Only data explicitly reported in the original publications or obtained directly from study authors will be included in the quantitative synthesis. Studies for which essential outcome data cannot be obtained will be retained for qualitative description only. This approach enables us to systematically organise and evaluate the relevant information extracted from the included documents, thereby enhancing the clarity and conciseness of our analysis. Additionally, any discrepancies that arise during the data extraction process will be resolved through discussion, and a third reviewer (GHH) will be consulted if necessary. The final extracted dataset will be securely stored for analysis.

#### 2.5.3 Risk of bias and quality assessment

To ensure the quality of the selected literature, this review will employ the critical appraisal tools of the Joanna Briggs Institute (JBI), the Cochrane Risk of Bias 2.0 tool, and the Grading of Recommendations, Assessment, Development and Evaluation (GRADE) system to assess the overall quality of the evidence (39–41).

The methodological quality of included randomised controlled trials will be assessed using the JBI Critical Appraisal Checklist for Randomised Controlled Trials. In the JBI evaluation, two reviewers (YYL and JFS) independently assessed the quality of the studies and resolved any differences by making consensus-based decisions or, if necessary, by discussion with a third reviewer (GHH). The scoring criteria will adopt the scoring system provided by the JBI guidelines to assess the quality of the included literature. The system assigns 1 point to each criterion that is fully met, with 1 point awarded for a “yes” answer and 0 points for a “no” or “unclear” answer. More than 80 percent of the total score is considered high quality, between 50 and 80 percent is considered medium quality and less than 50 percent is considered low quality. The appraisal results will be used to identify potential sources of bias and to inform the interpretation of findings. This systematic evaluation method ensures the reliability and scientific rigour of the review conclusions.

In the assessment of risk of bias, two reviewers (YYL and JFS) will independently evaluate each included study using the Cochrane Risk of Bias 2.0 tool. Discrepancies will be resolved through discussion, with consultation of a third reviewer (GHH) when necessary. Each study will be assessed across the five Risk of Bias domains, and judgements will be classified as “low risk of bias,” “some concerns,” or “high risk of bias” (42). An overall risk-of-bias judgement will be derived based on the domain-level assessments, in accordance with Risk of Bias guidance. Outcome measures will not be judged as high or low risk of bias solely on the basis of being subjective or objective. Instead, risk of bias in outcome measurement will be determined by factors such as blinding, susceptibility to influence by knowledge of the intervention, and consistency of outcome assessment across groups.

In the GRADE assessment, a comprehensive judgement is made from five dimensions: research bias, inconsistency, indirectness, imprecision and publication bias. If there are limitations, the evidence will be downgraded; if the results are consistent or the effect is significant, it can be upgraded. Ultimately, the evidence will be classified into four grades: high, medium, low, and very low (43). Two researchers (YYL and JFS) independently completed the scoring and calculated the K value to assess the consistency of the scoring, based on the Landis and Koch criteria: 0.00–0.20 indicates mild consistency, 0.21–0.40 indicates general consistency, 0.41–0.60 indicates moderate consistency, 0.61–0.80 indicates high consistency, and 0.81–1.00 indicates nearly perfect consistency. In case of any discrepancy, it shall be determined by the third researcher (GHH).

Importantly, studies scoring lower in JBI or GRADE assessments will not be excluded. We will explicitly highlight the methodological limitations of these studies within the systematic review and discuss their potential implications for the review’s conclusions.

### 2.6 Data synthesis and analysis

#### 2.6.1 Data synthesis

The statistical analysis of this study will be conducted using Stata 18.0 software (44). For continuous variables, the weighted mean difference (WMD) will be calculated when measurement methods were consistent across studies, or the standardised mean difference (SMD) when different measurement scales were used. For dichotomous variables, odds ratios (ORs) with 95% confidence intervals (CIs) will be computed. Heterogeneity among studies was assessed using the I^2^ statistic and Cochran’s Q test. Depending on the degree of heterogeneity, either a fixed-effect or random-effects model will be applied. Potential publication bias will be evaluated by Egger’s test and visual inspection of funnel plots. A *p*-value < 0.05 will be considered statistically significant.

#### 2.6.2 Heterogeneity assessment

The I^2^ statistic will be used to evaluate the degree of heterogeneity among studies. According to the Cochrane Manual, when I^2^ > 50%, significant heterogeneity is considered to exist (45). When *p* > 0.1 and I^2^ < 50%, the fixed effects model is adopted; When *p* < 0.1 and I^2^ > 50%, the random effects model will be adopted. If the data are sufficiently comparable, a meta-analysis will be conducted. If not comparable, the results will be reported using descriptive analysis methods.

In addition to statistical heterogeneity, potential sources of heterogeneity at the intervention and participant levels will be systematically examined. The following characteristics will be coded to capture intervention- and participant-level heterogeneity: (1) intervention duration, including total treatment length and cumulative exposure time; (2) type of VR system, categorised according to the technological configuration of the platform (e.g., immersive head-mounted display–based systems versus non-immersive or semi-immersive systems); (3) level of interactivity, classified as video-based exposure (passive viewing) versus interactive VR exposure involving user engagement or task-based interaction; and (4) participant characteristics, including baseline symptom severity and key demographic or clinical features when reported.

Intervention duration will be treated as a continuous variable when sufficient data are available, and categorised into higher versus lower exposure groups for subgroup analyses if necessary. VR system type and level of interactivity will be analysed as categorical variables. Participant characteristics will be examined to assess whether baseline clinical differences contribute to variability in treatment effects. Where sufficient studies are available, exploratory random-effects meta-regression analyses will be conducted for key continuous variables (e.g., intervention duration) to further investigate potential sources of heterogeneity.

#### 2.6.3 Sensitivity analysis

In this study, when significant heterogeneity is detected, leave-one-out sensitivity analysis will be used to evaluate the impact of individual studies on the overall results (46). Each study will be sequentially removed, and the pooled estimates and heterogeneity statistics will be recalculated. This procedure will help identify potential outliers and assess the robustness and stability of the meta-analytic findings.

#### 2.6.4 Publication bias assessment

In this meta-analysis, when a certain outcome measure involves 10 or more studies, potential publication bias will be evaluated through a funnel plot (47). The visual symmetry of the plots will be inspected to identify potential small-study effects or selective publication tendencies. To provide a quantitative assessment, Egger’s regression test will be performed at a significance level of 0.05 to examine funnel plot asymmetry, thereby enhancing the objectivity and statistical rigour of the analysis.

When fewer than 10 studies are included, formal statistical testing will not be conducted owing to insufficient power. Instead, a qualitative assessment will be performed based on study characteristics, sample sizes, effect directions, and the overall consistency of the findings. This comprehensive approach will facilitate a more reliable evaluation of potential publication bias and strengthen the robustness of the meta-analytic conclusions.

However, it should be noted that the ability to detect publication bias may be limited when the number of included studies is small or when relevant interventions are underreported. Under such circumstances, funnel plot–based methods and statistical tests may have reduced sensitivity, and the results of publication bias assessments will therefore be interpreted with caution.

## 3 Discussion

Consistent with prior reviews demonstrating the rapid growth of VR-based and self-guided digital interventions for anxiety disorders (34, 48, 49), existing systematic reviews and meta-analyses have primarily focused on therapist-guided VRET or CBT-based digital programmes (50, 51). As a result, the effectiveness of self-guided virtual reality exposure therapy (SGVRET) has not yet been systematically synthesised. The present review is designed to address this gap by systematically evaluating the effectiveness of SGVRET and synthesising available evidence across existing studies. However, several methodological challenges should be acknowledged. This review relies on intervention implementation details as reported in publicly available literature, and similar challenges have been widely documented in previous reviews of digital and VR-based mental health interventions. In particular, insufficient reporting of intervention components and implementation processes has been shown to limit reproducibility and hinder reliable evidence synthesis (52–54). Such limitations may affect the accurate identification and analysis of SGVRET intervention effects, potentially leading to the omission or misclassification of key intervention elements and, consequently, reduced reliability of pooled estimates. Moreover, high heterogeneity related to intervention design, technological platforms, implementation duration, study populations, and outcome measurement has been repeatedly identified as a major challenge in meta-analyses of digital mental health interventions (55–57). This heterogeneity may further constrain cross-study comparability and complicate quantitative synthesis. Previous methodological studies have also highlighted that publication bias is particularly difficult to assess in emerging digital interventions, where sample sizes are often small and reporting practices are inconsistent (58–60). By systematically examining methodological quality, sources of bias, and heterogeneity across existing studies, this review will address key methodological challenges identified in the current literature. This approach will enhance the interpretation of SGVRET intervention outcomes, highlight critical limitations and future research directions, and provide essential evidence for developing, evaluating, and implementing scalable self-guided virtual reality interventions for SAD.

## 4 Conclusion

This systematic review aim to address the current limitations in research concerning the efficacy of SGVRET in interventions for SAD. This systematic review will synthesise evidence from randomised controlled trials to assess the efficacy of SGVRET in alleviating social anxiety. Employing a rigorous methodological framework, the review will comprehensively evaluate SGVRET’s therapeutic efficacy, adherence rates, and clinical value for individuals with SAD. The expected research findings will provide robust theoretical insights and practical guidance for the design, implementation, and optimisation of future digital health interventions targeting anxiety-related symptoms. By synthesising high-quality evidence, this review will establish a solid foundation for subsequent SGVRET research and support the advancement of accessible, scalable, and clinically applicable digital exposure therapies.
