# Supplementary material for: Effectiveness of self-guided virtual reality exposure therapy for social anxiety disorder: a systematic review and meta-analysis protocol
Source: Front Public Health. 2026 Jan 12;13:1735471. doi: 10.3389/fpubh.2025.1735471 (PMC12832236; doi:10.3389/fpubh.2025.1735471)
Supplement: Supplementary file 1 [file Supplementary_file_1.docx]

**Supplemental Material 1. Search Strategies**

**PUBMED SEARCH STRATEGY**

(("Phobia, Social"[Mesh] OR "social anxiety" OR "social anxiety disorder" OR "social phobia" OR "public speaking anxiety" OR "social fear" OR "SAD") AND ("virtual reality exposure therapy" OR "virtual reality therapy" OR "VRET" OR "VR exposure" OR "virtual reality-based exposure" OR "immersive virtual reality") AND ("self-guided" OR "self-administered" OR "self-help" OR "self-led" OR "stand-alone" OR "automated" OR "self-care" OR "self-directed" OR "unguided" OR "therapist-free" OR "self-management" OR "digital self-help"))

**EMBASE SEARCH STRATEGY**

('social phobia'/exp OR 'social anxiety' OR 'social anxiety disorder' OR 'social phobia' OR 'public speaking anxiety' OR 'social fear' OR ('SAD' AND 'social anxiety')) AND ('virtual reality exposure therapy' OR 'virtual reality therapy' OR 'VRET' OR 'VR exposure' OR 'virtual reality-based exposure' OR 'immersive virtual reality') AND ('self-guided' OR 'self-administered' OR 'self-help' OR 'self-led' OR 'stand-alone' OR 'automated' OR 'self-care' OR 'self-directed' OR 'unguided' OR 'therapist-free' OR 'self-management' OR 'digital self-help')

**WEB OF SCIENCE SEARCH STRATEGY**

TS=((social anxiety OR "social anxiety disorder" OR "social phobia" OR "public speaking anxiety" OR "social fear" OR (SAD AND "social anxiety")) AND ("virtual reality exposure therapy" OR "virtual reality therapy" OR VRET OR "VR exposure" OR "virtual reality-based exposure" OR "immersive virtual reality") AND ("self-guided" OR "self-administered" OR "self-help" OR "self-led" OR "stand-alone" OR "automated" OR "self-care" OR "self-directed" OR "unguided" OR "therapist-free" OR "self-management" OR "digital self-help"))

**COCHRANE LIBRARY SEARCH STRATEGY**

("social anxiety" OR "social anxiety disorder" OR "social phobia" OR "public speaking anxiety" OR "social fear" OR ("SAD" AND "social anxiety")) AND ("virtual reality exposure therapy" OR "virtual reality therapy" OR "VRET" OR "VR exposure" OR "virtual reality-based exposure" OR "immersive virtual reality") AND ("self-guided" OR "self-administered" OR "self-help" OR "self-led" OR "stand-alone" OR "automated" OR "self-care" OR "self-directed" OR "unguided" OR "therapist-free" OR "self-management" OR "digital self-help")

**SCOPUS SEARCH STRATEGY**

TITLE-ABS-KEY((social anxiety OR "social anxiety disorder" OR "social phobia" OR "public speaking anxiety" OR "social fear" OR (SAD AND "social anxiety")) AND ("virtual reality exposure therapy" OR "virtual reality therapy" OR VRET OR "VR exposure" OR "virtual reality-based exposure" OR "immersive virtual reality") AND ("self-guided" OR "self-administered" OR "self-help" OR "self-led" OR "stand-alone" OR "automated" OR "self-care" OR "self-directed" OR "unguided" OR "therapist-free" OR "self-management" OR "digital self-help"))
