# Supplementary material for: Effectiveness of self-guided virtual reality exposure therapy for social anxiety disorder: a systematic review and meta-analysis protocol
Source: Front Public Health. 2026 Jan 12;13:1735471. doi: 10.3389/fpubh.2025.1735471 (PMC12832236; doi:10.3389/fpubh.2025.1735471)
Supplement: Supplementary file 2 [file Supplementary_file_2.docx]

**Supplementary Material 2.** Summary of Primary and Secondary Outcomes and Assessment Time

| **Author** | **Role** | **Outcome type** | **Outcome measure(s) (examples)** | **Expected time points** |
| --- | --- | --- | --- | --- |
| — | Primary | Social anxiety severity | LSAS / SIAS / SPS / etc. | Pre; Post; Follow-up (if reported) |
| — | Secondary (if included) | Public speaking anxiety | PSAS / etc. | Pre; Post; Follow-up (if reported) |
| — | Secondary (if included) | Fear of negative evaluation | BFNE / etc. | Pre; Post; Follow-up (if reported) |
| — | Secondary (if included) | Depression/General anxiety | PHQ-9 / BDI / GAD-7 / etc. | Pre; Post; Follow-up (if reported) |
| — | Secondary (if included) | Acceptability/Adherence | Dropout, completion, etc. | During intervention; Post |
| — | ... | ... | ... | ... |
